# Clinical experiences with a new endobronchial blocking device: the EZ-Blocker

**DOI:** 10.1186/cc9575

**Published:** 2011-03-11

**Authors:** T Végh, A Enyedi, I Takács, J Kollár, B Fülesdi

**Affiliations:** 1 University of Debrecen, Hungary

## Introduction

Both elective and emergency thoracic surgical procedures may require one-lung ventilation (OLV) for lung isolation [1]. Although in the majority of the cases a double lumen endotracheal tube (DLT) is the first choice, there are situations when insertion of DLT is not feasible [2]. We therefore intended to test the applicability of a recently developed endobronchial blocker (BB), the EZ-Blocker, in clinical practice.

## Methods

Data were obtained from 10 patients undergoing thoracic surgery necessitating OLV. For lung isolation, a single lumen tube (SLT) and EZ-Blocker as BB were used. The time of insertion and positioning of BB, the lung deflation time with the BB cuff inflated and deflated, the minimal occlusion volume (MOV) of the BB cuff with 25 cmH_2_O positive airway pressure (PAP) and intracuff pressure (ICP) at MOV were registered. Based on the CT scan the diameter of the right (RMB) and left main bronchus (LMB) at 1 cm distal apart from the bifurcation was measured offline. Lung deflation was defined as 5.5 cm distance of the upper lobe from the rib cage at open chest.

## Results

The insertion time was 76 ± 15 seconds. The lung deflation time through the lumen with the BB cuff inflated was 700 ± 83 seconds, and with a deflated cuff through the lumen of SLT was 9.4 ± 0.7 seconds. The MOV was 6.7 ± 1 ml in LMB versus 8 ± 1 ml in RMB (*P *= 0.03). The ICP was 40 ± 4 mmHg in LMB versus 85 ± 5 mmHg in RMB (*P *< 0.001). With linear regression there were strong positive relationships between the diameter of MB and MOV/ICP.

## Conclusions

The use of EZ-Blocker is easy and safe for infrequent users, too. The short insertion time and short lung deflation time allows use in an emergency situation or in case of a difficult airway. Only a small fraction of ICP (10 to 20%) is transmitted to the bronchial wall and it does not cause mucosal ischemia. The diameter of the MB has great impact on the MOV and ICP. The MOV is similar but ICP is smaller than published in previous reports with other BBs [3].
